# Feeding live Black Soldier Fly larvae (*Hermetia illucens*) to laying hens: effects on feed consumption, hen health, hen behavior, and egg quality

**DOI:** 10.1016/j.psj.2021.101400

**Published:** 2021-07-24

**Authors:** Fernanda M. Tahamtani, Emma Ivarsson, Viktoria Wiklicky, Cecilia Lalander, Helena Wall, T. Bas Rodenburg, Frank A.M. Tuyttens, Carlos E. Hernandez

**Affiliations:** ⁎ Department of Animal Nutrition and Management, Swedish University of Agricultural Sciences, Uppsala, Sweden; † Department of Energy and Technology, Swedish University of Agricultural Sciences, Uppsala, Sweden; ‡ Animals in Science and Society, Faculty of Veterinary Medicine, Utrecht University, Utrecht, The Netherlands; § Animal Sciences Unit, Flanders Research Institute for Agriculture, Fisheries and Food (ILVO), Scheldeweg 68, 9090 Melle, Belgium; # Department of Nutrition, Genetics and Ethology, Ethology and Animal Welfare Research Group, Faculty of Veterinary Medicine, Ghent University, Heidestraat 19, 9820 Merelbeke, Belgium

**Keywords:** laying hen, black soldier fly larvae, egg production, feed consumption, egg quality

## Abstract

The use of insects in animal feed has the potential to reduce the demand for soybean production and reduce the deforestation and loss of natural resources. In particular, the black soldier fly (**BSF**, *Hermetia illucens*) larvae have received attention due to their ability to convert organic waste into high-value biomass. Several studies have investigated the effects of providing BSF larvae to both broilers and laying hens. However, knowledge gaps regarding hens’ voluntary intake of live larvae and the effects of larvae consumption on egg production still remain. Therefore, the aim of the present study was to determine the effects of the consumption of 4 different amounts of live BSF larvae on laying hen feed consumption, hen health and fearfulness, and egg production and quality. To this end, 40 Bovans White laying hens were housed individually and provided with 0, 10, 20% or ad libitum daily portions of live larvae from 18 to 30 wk of age. The larvae consumption and concentrate consumption, hen weight, egg production, and egg quality were monitored. Overall, differences were found between the hens given ad libitum access to larvae compared to the other treatments. Ad libitum hens, consumed 163 ± 41 g larvae/hen/day, consumed less concentrate (*P* = 0.03) and gained more weight (*P* = 0.0002) than all other treatments. They also had an overall higher consumption of protein, fat and energy (*P* < 0.03). There was no effect of larvae consumption on egg production, egg weight, shell thickness, shell breaking strength, or Haugh unit (*P* > 0.05). There was also no effect on hen behavior toward a novel object or in an open field test. This study is the first to provide different amounts of live BSF larvae, including an ad libitum portion to laying hens. The 20% diet could promote sustainability in the egg industry and be economically advantageous if BSF larvae can be bought in bulk for less than 40% of the cost of the concentrate.

## INTRODUCTION

The human population is expected to reach 9 billion people by 2050 and this is likely to lead to food security and environmental issues (Alexandratos and Bruinsma, 2012). With this population increase, there is a growing demand for animal products and animal feed. For example, the production of layer and broiler feed saw a growth of 4 and 3%, respectively, during 2019 and the production of eggs increased from 15 to 87 million tonnes between 1961 and 2017 (Alltech, 2020; FAO, 2020). As agriculture and land use account for 25% of world greenhouse gas emissions (FAO, 2019) the use of insects for animal feed, as a substitute for soy products, has been recognized as a potential solution for this problem due to their nutritional value and lower environmental impact (Gasco et al., 2019). Furthermore, soy products for animal feed are largely exported from South American countries, where there are concerns with agriculture-associated deforestation and related human rights violations (Boerema et al., 2016). For example, a recent study suggests that 50% of the EU's carbon footprint from the import of Brazilian soybean is due to the deforestation of native forest and land use change implemented in that country to promote soybean farming (Escobar et al., 2020).

The use of insects in animal feed, therefore, has the potential to reduce the demand for soybean production and reduce the deforestation and loss of natural resources. In particular, the black soldier fly (**BSF**, *Hermetia illucens,* Diptera: Stratiomyidae) has received a lot of attention due to their larvae's ability to convert organic waste into high-value biomass and because they are not a disease vector as the adult fly does not eat (Sheppard et al., 2002; van Huis et al., 2013; Makkar et al., 2014). The larvae of the BSF are rich in protein and fat and thus, are a high-value feed source (Ewald et al., 2020; Giannetto et al., 2020). The use of insect-derived protein in poultry feed is not yet allowed under EU legislation (European Parliament, 2001). However, since the adoption of Regulation No 2017/893 authorizing their use in aqua feed, the European Commission is said to be exploring possibilities for proposing a revision which may authorize insect proteins in poultry feed as well (European Parliament, 2017; IPIFF, 2020).

Several studies have investigated the effects of providing BSF larvae to both broilers and laying hens. In broilers, BSF larvae meal is a good source of apparent metabolizable energy and digestible amino acids (De Marco et al., 2015; Schiavone et al., 2017b) and has been shown to improve growth rate (Oluokun, 2000). Feed containing BSF larvae oil has been shown to improve broiler chicken feed conversion ratio compared to corn oil and coconut oil (Kim et al., 2020). Furthermore, a substitution of soybean oil by BSF larvae fat supports performance, carcass traits, and overall meat quality (Schiavone et al., 2017a). In laying hens, the results are less consistent. For example, one study including full-fat dried BSF larvae in the feed resulted in an inferior feed conversion ratio and egg production compared to a control treatment that did not receive larvae (Bejaei and Cheng, 2018). Another study providing ad libitum dried larvae on the outdoor range found a reduction in egg weight, shell weight, shell thickness, and yolk color compared to control hens and found no differences between treatments in the ranging behavior of the hens (Ruhnke et al., 2018). In comparison, Kawasaki et al. (2019), found an increase in egg weight and egg shell thickness in a group fed dried whole BSF larvae compared to the control. A new area of interest is providing live larvae to laying hens, in order to further promote foraging behaviors and avoid abnormal behaviors such as feather pecking. Indeed, in a study of older laying hens, from 67 to 78 wk of age, the feather condition of live-larvae-fed hens was better than that of the control hens which were provided a commercial diet (Star et al., 2020). Furthermore, larvae provided throughout the day seemed to facilitate the expression of natural feed searching behavior without affecting feed conversion, body weight gain or egg parameters (Star et al., 2020).

However, while previous studies have provided chickens, both broilers and layers, with a known amount of larvae, those studies could not affirm that the birds ate all the larvae provided, particularly when larvae were fed live and could potentially escape from the birds. Furthermore, as the birds were always housed in groups, the larvae consumption could only be calculated as average per bird. This would not account for the effects of individual differences in levels of larvae consumption on egg production parameters, for example. In addition, while previous literature has substituted part of the daily feed ration with larvae, no study has yet provided the birds with ad libitum access to larvae, creating a significant knowledge gap regarding how much of this feed source chickens can and will eat and what effects this ad libitum access has on bird and production parameters. It is not known, for example, if layers can self-balance their feed intake when provided ad libitum access to larvae.

The aim of the present study was to determine the effects of the consumption of three specific and one ad libitum daily portions of live BSF larvae on laying hen feed consumption, health and behavior as well as egg production and quality. To this end, laying hens were housed individually and provided with 0, 10, 20% or ad libitum daily portions of live larvae from 18 to 30 wk of age. We hypothesized that hen weight would be proportionally positively affected and concentrate consumption would be negatively affected by larvae consumption. Furthermore, we expected that the provision of larvae might function as environmental enrichment and would, as such, reduce hen fearfulness in response to novel stimuli.

## MATERIALS AND METHODS

### Animals and Housing

The study included 40 Bovans White hens acquired from a commercial rearing farm (Närkesberg Hönseri AB, Åsbro, Sweden) at 16 wk of age. The hens were housed until 30 wk of age in a room (15.4 m × 10.8 m) of the experimental facilities at the Swedish Livestock Research Center, Lövsta. The room was equipped with 40 cages (75 cm × 48 cm × 150 cm H × W × L) with hens housed in pairs for the first 7 d and individually thereafter. The cages had solid floor and contained a nest box (33 cm × 30 cm × 30 cm H × W × L), a perch (30 cm), and wood shavings as litter. The cages allowed visual and auditory contact between the hens. The hens were allowed 2 wk of habituation to the experimental facilities, including feeding bowls and live larvae, prior to the start of the study, at 18 wk of age. During this habituation period, all hens were fed a commercial laying concentrate feed (Granngården Hönsfoder Värp, Sweden) and grit (Danshells, Denmark) ad libitum. Water was available ad libitum via 3 nipples per cage. On arrival, the light schedule was programmed for 24L: 0D for the first 24 h and thereafter 11L: 13D as recommended by the Bovans White Commercial Product Guide (Bovans, 2020). In the following weeks, the light hours were gradually increased until reaching 15L: 9D at 22 wk of age, which was maintained until the end of the study at 30 wk of age. The light intensity was approximately 10 lux and the temperature was kept at 21 to 24°C.

### Treatments

The 4 dietary treatments (10 hens/treatment) were:

1 Control/0%: No BSF larvae. Standard pelleted concentrate feed, optimized to meet the nutrient recommendation of the hens (Hendrix Genetics, 2020).
2 10%: Daily portion of live BSF larvae accounting for 10% of their daily expected DM- intake and a complementary concentrated pelleted feed.
3 20%: Daily portion of live BSF larvae accounting for 20% of their daily expected DM-intake and a complementary concentrated pelleted feed.
4 Ad libitum: Ad libitum access to live BSF larvae. Soy mash presented separately from the pelleted concentrate.

#### Diet Design

Full diet composition information is provided in Table 1. The diets and larvae were analyzed for dry matter by drying at 103°C for 16 h and ash was determined after ignition at 600°C for 3 h (Jennische and Larsson, 1990). The content of crude protein (N × 6.25) was determined by the Kjeldahl method (NMKL, 2003) and the ether extract was determined according to the European Commission directive establishing methods of analysis for determination of fats (European Commission, 1998). The respective concentrates for the 10 and 20% treatments were designed to fulfill the daily nutritional requirements of the hens when they receive 10 and 20% of their daily DM intake from the larvae. The concentrate for the ad libitum treatment was designed as for the 20% concentrate but without the addition soy, which was provided separately as mash. The proximate composition of the soy mash in dry matter basis was 1.4% crude fat, 52.5% crude protein, and 7.1% ash and apparent metabolizable energy was calculated as 10.4 MJ/kg DM (WPSA, 1989). The proximate composition of the larvae in dry matter basis was 25.8% crude fat, 46.7% crude protein, and 9.3% ash and apparent metabolizable energy was estimated as 16.60 MJ/kg DM (De Marco et al., 2015).

**Table 1:** Composition information for the concentrate pellets used in each treatment.

	Master mix			
Description	Amount %			
Limestone	46.3			
Wheat middlings	36.1			
Mono calcium phosphate	5.7			
Methionine	2.2			
Premix^1^	2.0			
Lysine	2.0			
Sodium bicarbonate	1.8			
NaCl	1.4			
Molasses	1.0			
Lignobond DD	1.0			
Valine	0.3			
Threonine	0.1			

	Amount % feed basis			
Treatment	10%	20%	Ad libitum	Control
Oats	11.8	13.3	14.2	10.0
Wheat	61.6	66.7	71.5	53.8
Soybean meal	14.1	6.7	-	17.4
Master mix	11.8	13.3	14.3	10.0
Rapeseed oil	0.4	-	-	2.7
NaCl	0.03	-	-	0.08
Limestone	0.4	-	-	6.0
Threonine	-	-	-	0.03
Valine	-	-	-	0.05

Analyzed proximate composition	Amount % DM basis			
Ash	9.2	9.4	9.8	14.1
Crude protein	17.2	13.9	12.3	16.8
Crude fat	2.6	2.8	2.3	4.5
Energy MJ ME/kg	12.2	12.4	12.5	12.0

The Master mix composition presented was the same for all concentrates.

1 Premix provided/kg feed: Xanthophyll yellow 4.5 mg; Xanthophyll red 5.5 mg; Phytas Danisco 400 FTU; Vit D3 3,000 IU; Vit K3 3 mg; Vit B5 9 mg; Folic acid 1 mg; Biotin 0.2 mg; Vit B3 Niacin 30 mg; Vit E 35 mg; Vit A 10,000 IU; Vit B1 2 mg; Vit B12 0.02 mg; Vit B2(5-phosphat) 5 mg; Vit B6 3 mg; T Fe (FeSO4) 20 mg; T Cu (CuSO4) 5.99 mg; T I (Ca(IO3)2) 2 mg; T Se (Na2SeO3) 0.35 mg; T Zn (ZnSO4) 80 mg; T Mn (MnSO4H2O) 70 mg.

All hens were provided ad libitum access to grit. Each component of the diet (i.e., concentrated pellets, soy mash, grit, and live larvae) was provided in dedicated feed bowls/troughs.

#### Black Soldier Fly Larvae Production

The larvae used for this experiment were produced and portioned at the Black Solider Fly colony of the Environmental Engineering group at the Department of Energy and Technology of the Swedish University of Agricultural Sciences (SLU, Uppsala, Sweden).

The production of the larvae took place in a greenhouse during the months of May to August 2020, with an average room temperature of 23°C and a relative humidity of 65%. The following feeding regime was used: the starter larvae (1 mg/larva) were reared in boxes (60 cm × 40 cm × 20 cm) and kept in racks of 11 boxes. Each box contained 12,000 larvae which equals to 5 larva/cm^2^. The applied feed was calculated so each larva received 0.2 g volatile substance/larva of poultry feed throughout the growth period. The feed pellets were watered down with 1:2 parts of water to achieve a feed containing 30% dry matter. The poultry feed used was leftover feed provided by SLU's experimental farm in Lövsta, Sweden. The feeding was split into 4 feedings during the larval growth period. The larvae were harvested before 5% of the larvae turned into prepupa, to ensure that the majority of the larvae were at the same stage of development. This estimation ensured a similar body composition of all the larvae in terms of protein and fat content.

An analysis of the DM content of the larvae was performed prior to the start of the study and showed an average 32% DM. This DM content was used to calculate the daily portion of larvae the total estimated DM intake of 100 g/day allocated to the hens in the 10% (31 g larvae/hen/day) and 20% (63 g larvae/hen/day) treatments for the duration of the study. The initial portion allocated to the hens in the ad libitum group was the equivalent of 40% (125 g larvae/hen/day). This portion was gradually increased until a true ad libitum provision was achieved (i.e., when the hens consistently did not eat all larvae in the portion within 24 h) at 21 wk of age. The daily larvae portions were prepared at the BSF facilities and transported to the experimental facilities at Lövsta twice a week, with larvae being weighed according to each portion size and boxed in transparent rectangular plastic boxes (175 mm × 120 mm × 40 mm; 500 mL; art nr F500, Tingstad). Holes were drilled into the lids of the boxes to allow air circulation. After portioning, the boxes were transported to the experimental facilities at Lövsta and kept in a cold room (approx. 15°C) until use (1–4 d later). The live larvae were presented to the hens in non-spill bowls designed for dogs (18.5 cm × 8 cm; 1.4 L). These bowls had a lid with a 4.2-cm wide brim which barred the larvae from crawling out of the bowl while still allowing the hens to access the larvae inside.

### Data Collection

#### Feed Consumption and Hen Weight

The larvae consumption was monitored daily, with the weight of leftover larvae from the last 24 h being noted before being discarded and a new daily portion being provided. When necessary, the bowls were cleaned before the new portion was placed inside. The weight of the leftover larvae was controlled for the loss of weight of the larvae due to exposure to the open air of the chicken room. This was calculated with 4 larvae bowls per larvae portion size provided to the hens (e.g., 31 g, 63 g, 125 g,) being left inside the chicken room, so to expose them to the same temperature and ventilation, and the weight of the portion being measured after 24 h. From this, an average weight loss per larvae portion size was calculated. The consumption of the other components of the diet (i.e., concentrated pellets, soy mash, and grit) was monitored weekly. This was calculated by comparing the weight of the feed containers at the start and the end of the week. The weight of the hens was also measured weekly, on the same day as the weight of the feed.

#### Egg Production and Quality

Egg production was monitored daily for the first 2 wk of the experiment (wk 18 and 19 of age) and again for the last 6 wk of the study (wk 25 to 30 of age). In addition, the weight of the eggs was measured 3 times a week.

Egg quality was monitored every 2 wk. The following parameters were assessed: egg weight was measured using a Kern PCB balance with ± 0.01 g accuracy. Eggshell thickness (mm) was measured using a digital Mitutoyo absolute thickness gauge and calculated as the mean of 3 measures from the equator of the egg. Eggshell weight (g) was measured using the Kern PCB balance after the eggshell had been washed and left to dry overnight at room temperature. Yolk color was measured using a Roche yolk color fan. Eggshell breaking strength (kgF) was measured using an Egg Force Reader (Orka Food Technology Ltd., West Bountiful, UT). Egg white/albumen height (mm) was measured using an Ames s-6428 micrometer with 0.1 mm accuracy. Yolk weight (g) was measured using the Kern PCB balance after the yolk had been separated from the white. The weight of the egg albumen was calculated from the egg weight minus the combined weight of the yolk and the eggshell. The albumen height, yolk weight, and shell breaking strength were measured only on wk 26, 28, and 30 of age. Finally, Haugh unit was calculated using the following formula where HU = Haugh unit, h = albumen height (mm), w = egg weight (g):

$$HU=100*\log10\left(h-1.7w^{0.37}+7.6\right)$$

#### Open Field Test

At 29 wk of age, the birds’ behavioral response in a novel open field was video recorded. The field consisted of a 1 × 1 m arena with 60 cm of solid walls and 70 cm of wire mesh walls above the solid walls. The top was partly covered by wire mesh to prevent birds from escaping, while still providing a clear image of the arena for the video camera installed above. The arena was located in an adjacent room where birds could not hear or see any other birds. Individual birds were transported from the home pen in the arms of the experimenter to the arena room (lights turned off at placement to prevent birds from escaping) and placed in the middle of the arena. The test lasted 10 min starting immediately after the experimenter left the room and had turned the lights on. The birds were returned to their home pen immediately after the test. The arena was cleaned of any droppings before starting the next test. From the video recordings, an observer blind to the treatment of the birds recorded the time spent pacing, which has been associated with fear in this test (Jones, 1982; Suarez and Gallup, 1983). The performance of gakel calls, escape attempts and fecal droppings during the open field test were each scored on a dichotomous scale (Yes/No). Gakel calls and fecal droppings are often assessed during open field tests and have been associated with frustration and fear in laying hens (Jones, 1982; Jones and Merry, 1988; Zimmerman et al., 2000).

#### Novel Object Test

At 30 wk of age, the birds’ behavioral response to a novel object (**NO**) in the home cage was video recorded for 10 min. Two objects were used, one for the odd numbered cages and another for the even numbered cages. This was done to avoid birds habituating to the NOs during the testing of birds in adjacent cages. The 2 objects were a wooden colored stick (50 cm long and 2 cm in diameter) and a 500 mL orange bottle (23 cm long and 6 cm in diameter). A video camera was placed on a tripod in front of the cage and the NO was placed inside the cage on the front right corner, in front of the nest box. No personnel was present inside the room during the video recording. Later, an observer scored the videos. Observations started 30 s after the start of the recording, to allow sufficient time for the researcher to exit the room, and continued for 9 min. The observer scored the following behaviors of the birds from the videos: time spent in the half of the cage closest to the NO, did the bird touch the NO (Yes/No) and the latency to touch the NO, which have been associated with fear in this test (Jones, 1985; Forkman et al., 2007).

#### Postmortem Assessment

At the end of the experiment, at 31 wk of age, a postmortem assessment was performed on all hens. The hens were killed with an intravenous injection of pentobarbital (Allfatal vet. 100 mg/mL. Omnidea AB, Stockholm). After death, the weight of the following organs was recorded: empty proventriculus, empty gizzard, liver, and abdominal fat pad.

### Ethical Statement

All procedures involving animals were approved by the ethical committee of the Uppsala region of the Swedish Board of Agriculture (Jordbruksverket), application number 5.8.18-03402/2020.

### Statistical Analysis

Statistical analyses were performed using the software SAS 9.4. Data from one hen in the ad libitum larvae treatment were excluded from the analysis due to a cessation in egg laying at 24 wk of age, body weight loss, and egg yolk peritonitis. In addition, one hen from the 10% treatment died unexpectedly at 26 wk of age. Nevertheless, the data collected from this hen prior to her death was included in the analysis. When analyzing the interaction between treatment and week of age, for all dependent variables, week of age was treated as a continuous variable while treatment and cage were treated as categorical variables to avoid the large number of pairwise comparisons during post hoc analysis. When deemed necessary, pairwise comparisons between treatments during a specific week of age were performed. The critical *P*-value associated with these analyses was Bonferroni corrected to α = 0.008 (i.e., 6 pairwise comparisons between treatments within week of age).

The results of the larvae consumption are presented as descriptive statistics, with means and standard deviation, as the live larvae weight consumed per hen per day. The data collected on hen weight, total concentrate consumption (i.e., all pellets, soy and grit but not including the live larvae), egg laying percentage, and egg quality were analyzed using the mixed procedure with treatment and week of age, as well as their interaction, as fixed factors. The model for the analysis of egg shell thickness also included the whole weight of the egg as a covariate. Cage was included in the models as a random factor. Where appropriate, post-hoc analysis was performed with the Tukey test (Tukey's HSD test). Due to technical issues, the amount of grit consumed in wk 18 of age was not included in the analysis. Total consumption of crude protein, crude fat, and energy (MJ ME) was calculated per week and analyzed as described above. Due to an error in the control of the feed consumption during wk 23 the data for this week were excluded from the analysis of the energy, protein, and fat consumption.

The data on the time spent close to the NO were analyzed using the mixed procedure, with treatment as a fixed effect and NO type as a random effect. None of the hens touched the NO during the NO test. Therefore, these data and the latency to touch the NO were not analyzed. The data on the time spent performing pacing behavior in the open field test were analyzed using the mixed procedure, with treatment as a fixed effect. The model residuals were not normally distributed and were log transformed to fit the assumptions of the model. The performance of gakel calls and fecal droppings during the open field test were analyzed using a binary glimmix procedure with treatment at a fixed effect. The performance of escape attempts could not be statistically analyzed due to low occurrence.

The weight of the gizzard, proventriculus, liver, and abdominal fat, where analyzed using the mixed procedure with treatment as the fixed factor. In addition, the live body weight of the hens was included in the model as a covariate.

## RESULTS

**Figure 1:**
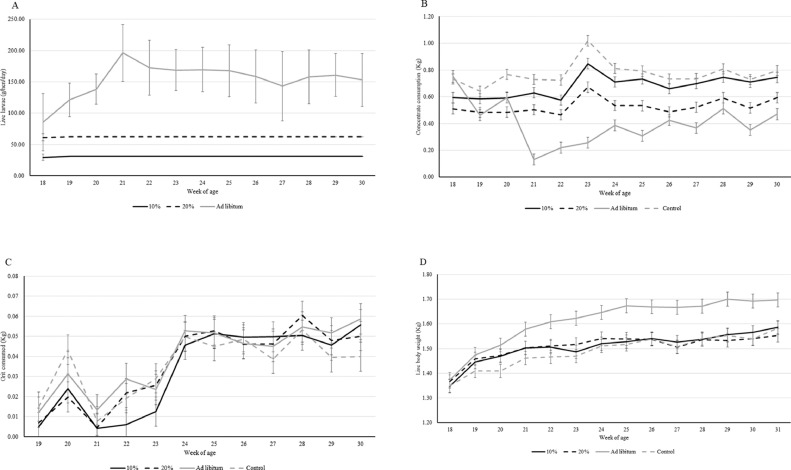
LS mean ± SE larvae consumption (panel A), concentrate consumption (panel B), grit consumption (panel C), and live bodyweight (panel D) across week of age for each treatment.

The hens in the 10 and 20% treatments readily ate all of the larvae in the daily portion, never leaving any larvae behind after the first week of the study (Figure 1A). Ad libitum hens consumed an average of 163.1 ± 41.6 g live larvae/hen/day (range: 35.6–235 g; i.e., approximately 52% of estimated DM intake).

There was an effect of the interaction between treatment and week of age on the total amount of concentrate consumed (F_3,458_ = 5,79; *P* = 0.0007), where the concentrate consumption in the ad libitum hens decreased after wk 21 of age, as compared to the other treatments (*P* < 0.03). Furthermore, most concentrate was consumed by the control hens, while the smallest amount of concentrate was consumed by the ad libitum hens (Figure 1B). The amount of concentrate consumed by the hens in the 20% group was somewhat stable throughout the experiment and intermediate between that of the ad libitum hens and the 10% and control hens.

There was an effect of the interaction between treatment and week of age on the consumption of grit (F_3,418_ = 4.55; *P* = 0.004), where the control treatment started with a higher consumption and ended with a smaller consumption than that of the other treatments (*P* < 0.05; Figure 1C). With regards to hen weight, there was an interaction effect between treatment and week of age (F_3,497_ = 20.26; *P* < 0.0001; Figure 1D) where the ad libitum hens had a steeper growth curve and reached a higher body weight than the hens of the other treatments (*P* < 0.0002). In addition, the 10% and control hens had steeper growth curves compared to hens from the 20% treatment (*P* < 0.01).

**Figure 2:**
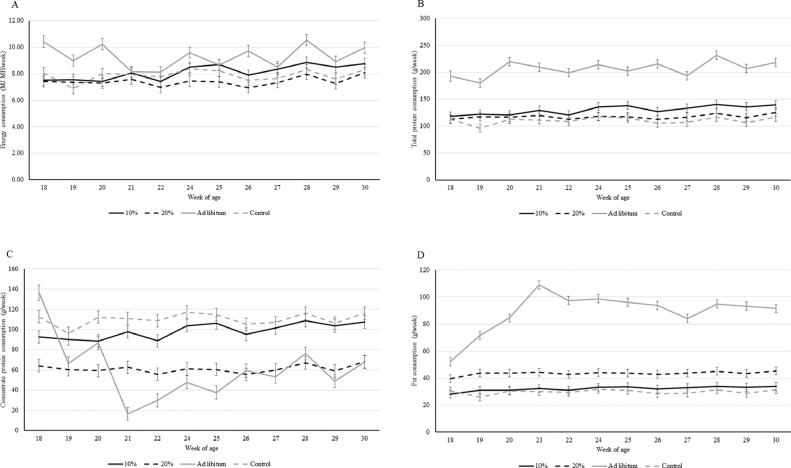
LS mean ± SE energy consumption (panel A), total protein consumption (panel B), concentrate protein consumption (panel C), and fat consumption (panel D) across week of age for each treatment.

There was an effect of the interaction between treatment and age on the total energy consumed (F_3,419_ = 3.08; *P* = 0.03). As can be seen in Figure 2A, the ad libitum hens seemed to consume more energy than the hens of the other treatments in some weeks, particularly at the start and end of the study. At 30 wk of age, for example, the ad libitum hens consumed more energy than the hens in the 20% and control treatments (*P* < 0.006) but did not differ from the 10% hens (*P* = 0.04; Bonferroni corrected α = 0.008).

There was an effect of the interaction between treatment and week of age on the total protein consumption (F_3,418_ = 2.88; *P* = 0.03; Figure 2B). Overall, the hens in the ad libitum group consumed more protein than the hens from the other treatments (*P* < 0.0001), and this consumption increased slightly from approximately 190 g/week at the start of the study, to approx 220 g/week at 30 wk of age. When we consider the protein consumption from the concentrate feed only, there was an interaction effect between treatment and week of age (F_3,418_ = 4.70; *P* = 0.003). The ad libitum hens had a high consumption of protein from the concentrate (including the soy mash) at the start of the study, which then declined sharply until wk 21, when the true ad libitum portion of live larvae was reached (Figure 2C). Thereafter, their concentrate protein consumption increased until the end of the study, reaching levels similar to that of the hens in the 20% treatment (*P* = 0.94) and significantly higher than that of the 10% and control hens (*P* < 0.0001).

With regard to the consumption of fat, there was an effect of the interaction between treatment and week of age (F_3,422_ = 11.31; *P* < 0.0001). As shown in Figure 2D, the fat consumption by the ad libitum hens increased during the first weeks of the study and was higher than that of the hens in the other treatments (*P* < 0.0001).

**Figure 3:**
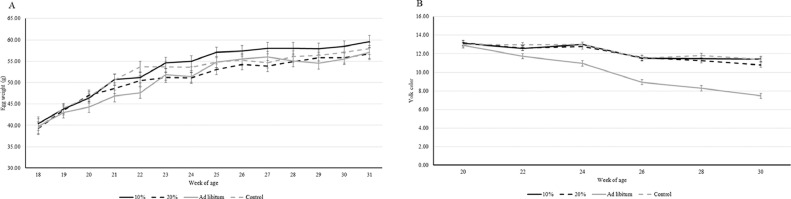
LS mean ± SE egg weight (panel A) and yolk color (panel B) across week of age for each treatment.

There was no effect of treatment on the laying percentage (F_3,34_ = 1.73; *P* = 0.18). As expected, the laying percentage increased with age (F_8,276_ = 72,21; *P* < 0.0001). The hens started laying at 18 wk of age with a laying percentage of 24.17 ± 31.80% (means ± std dev). When the experiment ended, at 30 wk of age, the laying percentage was 97.36 ± 5.61%. With regards to the weight of the eggs, there was no effect of treatment (F_3,469_ = 0.97; *P* = 0.40). There was an effect of age on the weight of the eggs (F_1,1580_ = 1329.40; *P* < 0.0001), with the egg weight increasing in the early weeks of lay (Figure 3A).

There was no effect of treatment on egg shell thickness, shell breaking strength, Haugh unit, height of the albumen, shell percentage, yolk percentage, or white percentage (Table 2). There was, however, an interaction effect between treatment and week of age on the color of the egg yolks (F_3,173_ = 35.86; *P* < 0.0001), in that the eggs laid by ad libitum hens had a sharper decline in the yolk color score; that is, the yolks became lighter in color as the hens got older compared to the eggs laid by hens in the other treatments (*P* < 0.0001; Figure 3B).

**Table 2:** Results for egg quality parameters (mean ± std dev and test statistics for the effect of treatment).

Parameter	Mean	Std dev	Test statistic
Shell thickness*	0.38 mm	0.02 mm	F_3,35_ = 1.26; *P* = 0.30
Shell breaking strength	5.32 kgF	0.85 kgF	F_3,34_ = 1.47; *P* = 0.24
Haugh unit	100.15	4.37	F_3,35_ = 1.69; *P* = 0.19
Albumen height	9.97 mm	0.99 mm	F_3,34_ = 0.77; *P* = 0.52
Shell percentage*	10.40%	0.64%	F_3,35_ = 0.75; *P* = 0.52
Yolk percentage*	25.14%	1.56%	F_3,34_ = 1.65; *P* = 0.19
Albumen percentage*	64.61%	2.0%	F_3,34_ = 0.86; *P* = 0.47

⁎ Means± std dev presented are from 30 weeks of age.

There was no effect of treatment on the time spent close to the NO (F_3,33_ = 0.66; *P* = 0.58), with all birds spending an average of 23.8% of the observation time in the half of the cage close to the NO (LS Means ± SE: 128.71 ± 56.35 s). Nor was there an effect of treatment on the duration of pacing behavior in the open field test (F_3,34_ = 0.07; *P* = 0.97), with birds from all treatments spending an average of 8.85 ± 6.09 s performing pacing behavior. There was no effect of treatment on the performance of gakel calls (F_3,34_ = 0.64; *P* = 0.59) or of fecal droppings during the open field (F_3,34_ = 0.08; *P* = 0.97). Only one of the hens, from the ad libitum treatment, performed an escape attempt during this test.

The ad libitum hens had significantly heavier abdominal fat pads and proventriculi compared to the other treatments, whereas the 10, 20%, and control treatments did not differ from each other (Table 3). There was a tendency for the ad libitum hens to have lower liver weight and the 10% hens to have higher liver weight (*P* = 0.054). Finally, there was no effect of treatment on the weight of the gizzard.

**Table 3:** Weight of the different organs and live hen bodyweight at 31 weeks of age (LS means (g) and standard error) across treatment along with the test statistics for the effect of treatment.

Organ	Weight ± SE (g)				Test statistic
	10%	20%	Ad libitum	Control	Treatment
Proventriculus	7.65 ± 0.36^b^	7.66 ± 0.35^b^	9.34 ± 0.39^a^	7.72 ± 0.34^b^	**F_3,33_ = 4.49** ***P* = 0.009**
Gizzard	22.68 ± 1.00	23.30 ± 1.02	23.21 ± 1.10	23.91 ± 0.95	F_3,32_ = 0.27 *P* = 0.84
Liver	54.21 ± 1.95	50.91 ± 1.89	45.73 ± 2.12	50.06 ± 1.85	F_3,33_ = 2.82 *P* = 0.054
Abdominal fat	40.95 ± 3.86^b^	47.22 ± 3.75^b^	68.56 ± 4.20^a^	39.74 ± 3.66^b^	**F_3,33_ = 10.31 *P* < 0.0001**

Live hen bodyweight	1,585 ± 37^ab^	1,553 ± 35^a^	1,697 ± 37^b^	1,583 ± 35^ab^	**F_3,34_ = 3.03** ***P* = 0.04**

a-b Different letters and bold text within explanatory variable indicate significantly different values (*P* < 0.05).

## DISCUSSION

The present study investigated the effects of four dietary treatments differing in the content of live black soldier fly larvae on feed consumption, hen health and behavior, and egg quality. Hens given ad libitum access to live larvae voluntarily consumed 163.13 ± 41.63 g live larvae/hen/day, consumed less concentrate, gained more weight, had heavier proventriculi and had more abdominal fat compared to hens fed restricted amounts of larvae or a control diet. In addition, ad libitum hens laid eggs with paler yolks than the hens from the other treatments. No differences between treatments were found in the other analyzed parameters such as egg production, egg weight, egg shell thickness, weight of the gizzard, and liver or fearfulness.

Larvae are readily consumed by chickens in natural conditions and are considered a palatable feed (Mench, 2009). Indeed, insect larvae, such as mealworms (*Tenebrio molitor*), are often used as reward for example in chicken studies that use operant conditioning paradigms or to encourage the performance of certain behaviors (Moe et al., 2014; Tahamtani et al., 2015). As expected, the hens in the present study showed great interest in the larvae. Anecdotal observations during feeding time showed that the hens in the 10 and 20% treatment groups would consume their entire portion of live larvae in approximately 5 min. When provided ad libitum, the hens would not eat so voraciously, rather spacing out consumption across the hours of the day, potentially due to a decrease in the viewed value of the larvae as they were always available. Indeed, the hens consumed most larvae in the first week of ad libitum provision and thereafter larvae consumption decreased slightly, likely reflecting a decrease in the demand for the larvae as the supply was more than sufficient. The amount of larvae consumed by the ad libitum hens also puts into context the amounts provided in other studies. For example, Star et al. (2020) provided 12 g of live larvae/hen/day which accounted for just 7% of the ad libitum consumption of the hens in the present study. It is also important to note the large standard deviation in the consumption of larvae of the ad libitum group. This points to a large individual variation in larvae consumption. This is very relevant for studies that provide larvae to a group of chickens, as it can be expected that not all individuals will consume their fair share of larvae. An unequal consumption of larvae would be further exacerbated by the competition for the larvae and the rank order of the hens in a group (Shimmura et al., 2008).

The concentrate consumption was largely complementary to the larvae consumption. The ad libitum hens consumed less concentrate than the hens from the other treatments, particularly after wk 21 of age when true ad libitum was reached for the daily larvae portion. In addition, the 20% hens also consumed less concentrate than the 10% and control hens, suggesting that the hens replaced the concentrate feed for the larvae and did not supplement their concentrate consumption with large amounts of live larvae. This is highly relevant in the mission to increase the sustainability of egg production by reducing the need for less sustainable feed components, such as soy products. Feed production accounts for 25.5% of the CO_2_ emissions generated in chicken meat and egg supply chains (i.e., 606 million tones CO_2_/year) (MacLeod et al., 2013). In addition, as soy products are imported, largely from South American countries such as Brazil, Argentina, and Paraguay (IDH and IUCN NL, 2019), the transatlantic transport of soy products increases the carbon footprint, further reducing the sustainability of egg production. A recent study has shown that 5% of the European carbon footprint related to imported Brazilian soybean is due to maritime transport (Escobar et al., 2020). Furthermore, due to a closer geographical proximity to EU ports, the soybean imported from Brazil originates from Northern Brazil, which is a hotspot of deforestation for agriculture (Escobar et al., 2020). As a result, this deforestation/land use change accounts for more than 50% of the EU's carbon footprint in the Brazilian soybean supply chain (Escobar et al., 2020). There is, therefore, a large potential for reducing the emission of greenhouse gases in the poultry production chain if locally produced larvae are fed organic waste and introduced to the feed as a protein alternative. As shown in the present study, the overall consumption of fat, protein, and energy did not differ between the hens in the 10, 20, and control treatments. Furthermore, there were no differences between these three treatments in hen body weight, egg production, egg quality, or hen health. There was, however, a reduction of 25% of concentrate consumption in the hens in the 20% treatment, suggesting that this level of larvae inclusion in the diet can have positive effects in increasing the sustainability of egg farming without compromising egg production, egg quality or hen health. Further studies would be necessary to verify whether a higher level of larvae provision, for example, 30 or 40%, would also result in no effects on hen performance but further reduce the consumption of concentrate.

An ad libitum access to live larvae, however, is wasteful as these hens consumed more protein than necessary. The results from the analysis of protein consumption showed that, by the end of the experiment at 30 wk of age, the ad libitum hens where consuming approximately 102 g/week more protein than the control hens, and that 69% of their total protein consumption was due to the consumption of larvae. This diet would, consequently, likely result in nitrogen leakage into the environment from the manure and potentially lead to pollution of the ground and water (Nahm, 2002). In comparison, the hens from the 20% treatment consumed only 9 g/week more protein than the control hens at 30 wk of age, and 45% of their total protein consumption came from the larvae. Therefore, it is likely that an inclusion of 20% of live larvae to the diet of laying hens would not result in a considerable increase in nitrogen leakage.

The results on the concentrate and larvae consumption are also in line with the observed increase in body weight of the ad libitum hens compared to the other treatments. It must be noted that the concentrate used in the ad libitum treatment was designed as for the 20% concentrate but without the addition of soy, which was provided separately as mash. This was done because the diets were designed before the size of the ad libitum consumption of larvae was known. This may have exacerbated the weight gain of the hens in the ad libitum treatment in this study. Nevertheless, as seen in the postmortem assessment, the ad libitum hens had more abdominal fat than the hens in the other treatments, due to the relatively high fat content of the larvae and their higher overall fat consumption compared to the hens in the other treatment groups. While the statistical analysis in this study showed that the ad libitum hens consumed more energy than the hens in the other treatments, this difference was not as large as expected based on the results of the weight of the abdominal fat and fat consumption. As the energy content of the live larvae in the present study was taken from De Marco et al. (2015), these results may suggest that these adopted values were not a good estimate for the larvae used here. This, in turn, highlights a need for methods for estimating the energy value of insect larvae, much as the European Table of Energy Values for Poultry Feedstuffs is used for other ingredients (WPSA, 1989). An important point to consider, nevertheless, is that the hens in the present study were housed individually in cages and therefore, had an arguably sedentary life compared to a non-cage housing system. Thus, it is possible that some of this increase in body and abdominal fat weight observed in the ad libitum hens would be curbed if the hens had been housed in an environment that allowed more physical activity (Bari et al., 2020). Still, the overwhelming majority of the nearly 7.5 billion laying hens alive today are housed in cages (Schuck-Paim et al., 2021) and, therefore, the results presented here are also relevant to them.

The postmortem analysis showed that the ad libitum hens also had heavier proventriculi. The proventriculus is the glandular part of the stomach of birds that stores and commences the digestion of food by the secretion of hydrochloric acid and pepsin (Klasing, 1998). This organ also contracts to provide adequate mixing between the food and the digestive enzymes before the gastric contents move on to the gizzard for further grinding (Oglesbee, 2006). The results, therefore, suggest an increased load on the proventriculus when digesting a larvae rich diet. Indeed, it has been suggested that the chitin present in the exoskeleton of insects is not easily digested by domestic poultry (Ravindran and Blair, 1993). Interestingly, the same effect was not observed on the gizzard. This might indicate that the larvae introduced an increased need for enzymolytic action (e.g., increased need for pepsin with the higher protein consumption) rather than mechanical action. Further studies are needed to support this hypothesis.

The high fat content of the larvae was expected to lead to an excess in energy consumption and, in turn, cause an accumulation of fat in the liver. However, the liver of ad libitum hens tended to weigh less than those of the hens from the other groups. It is possible that the ad libitum consumption of larvae had an effect on the metabolism of the hens. However, further research is needed to verify this. Still, it is important to point out that the present study had a short duration relative to the full production cycle of laying hens. At the end of this study, the hens were 31 wk of age and, as such, expected to be still in very good physical condition. It remains to be seen what would be the effects of these diets if provided for the whole laying period (i.e., until approx. 75–80 wk of age). Potentially the weight difference between the ad libitum hens and the others would continue or perhaps even increase and the adverse effects of a high fat diet might become more visible.

There was no effect of any of the diet treatments on egg production, egg weight, or most of the egg quality parameters investigated. The only exception was the color of the egg yolks, which was found to be paler in the ad libitum hens. This effect is likely explained by a reduced consumption of carotenoids (e.g., xanthophyll), which were provided by natural raw materials in the concentrate (Table 1). Carotenoids exert antioxidant effects and have known benefits to human health such as improving eye health, cardiovascular health, cognitive function and may even help prevent some types of cancer (see review by Eggersdorfer and Wyss, 2018). Consumers have been shown to give considerable importance to the color of egg yolks, even over other egg characteristics such as texture, flavor, and odor (Berkhoff et al., 2020). In order to ensure a strong pigmentation of the egg yolks in a larvae rich diet it would be necessary to increase the provision of carotenoids in the concentrate or, perhaps, in the larvae (by supplementing their diet).

As previously mentioned this study covered only the initial part of the laying period and was terminated when the hens were at the peak of lay. A recent study provided 12 g of live BSF larvae per hen per day, on top of a soy-free concentrate, to laying hens and found no effects on egg shell breaking strength, shell elasticity or Haugh unit (Star et al., 2020). However, this diet was provided only at the end of the laying period, from 67 to 78 wk of age. Therefore, further studies are needed to determine the effects of allocations of larvae to laying hens on the egg production and egg quality parameters when provided for the entire laying period.

There was no observed effect of any of the diets on hen fear and frustration behavior in response to novel stimuli, as assessed in the novel object and open tests (Jones, 1982; Zimmerman et al., 2000; Forkman et al., 2007). These results show that the simple provision of larvae in a bowl is not enough to function as environmental enrichment and affect hen behavior. Alternatively, the larvae provision may affect hen behavior and/or affective states in ways other than via fear. Nevertheless, as the larvae are indeed highly valuable to the hens, there is great potential in presenting the larvae in ways that can function as environmental enrichment, for example by scattering the larvae in the litter to stimulate the performance of natural behaviors, such as foraging, while reducing the incidence of maladaptive behaviors such as feather pecking and reducing fearfulness (Nørgaard-Nielsen et al., 1993; Tahamtani et al., 2016; Star et al., 2020).

Finally, it is important to discuss the economic cost/benefit of introducing live BSF larvae into the diet of laying hens. In the present study, a provision of 20% of live larvae had no negative impact on hen performance. Therefore, feed cost would arguably be the parameter of most interest to deduce a net margin. Based on the present results showing a 25% reduction in concentrate consumption in the 20% hens, it is possible to calculate that if the 20% concentrate comes to retail at the same price as the conventional concentrate, 1 kg of live BSF larvae would have to cost less than 40% of the cost of 1 kg of concentrate for this diet to result in a net gain compared to the conventional diet.

## CONCLUSIONS

The present study is the first to house laying hens individually and provides different amounts of live BSF larvae, including an ad libitum portion. This study design allowed for the careful monitoring of all aspects of larvae and concentrate consumption of each hen, as well as any effects of the diets on egg production and egg quality. The results of the study provide valuable context to any past or future studies where hens are presented with a portion of their daily feed as larvae, particularly with regards to how many larvae they would consume if given ad libitum access. The results also indicated that while a substitution of 10% of the conventional feed for live larvae was not enough to reduce concentrate consumption, a substitution of 20% decreased concentrate consumption by 25%. Furthermore, the overall consumption of fat, protein and energy did not differ between the hens in the 10, 20%, and control treatments. These are important findings to consider when implementing insects in laying hen feed with the purpose of reducing the use of plant protein such as soy.
